# Editorial: Epigenetics in prostate cancer

**DOI:** 10.3389/fonc.2023.1268519

**Published:** 2023-11-07

**Authors:** Aria Baniahmad, Mustafa Ozen

**Affiliations:** ^1^ Institute of Human Genetics, Jena University Hospital, Jena, Germany; ^2^ Department of Pathology & Immunology, Baylor College of Medicine, Houston, TX, United States; ^3^ Department of Medical Genetics, Istanbul University Cerrahpasa-Medical School, Istanbul, Türkiye

**Keywords:** lncRNA, chromatin architecture, immune escape, anoikis, higher order chromatin architecture, prostate cancer, tumor microenvironment

Epigenetic pathways include a broad spectrum of regulatory mechanisms that are part of tumor evolution (1, 2). Epigenetics participates in many aspects in the control of prostate cancer (PCa) tumorigenesis, presumably to provide a rapid mechanism with high flexibility in response to the environment. This includes control of cancer growth, metastasis, the development and maintenance of therapy resistance, immune escape, and cell–cell interaction in the tumor microenvironment, as well with the immune system, but it also includes programmed cell death of cancerous cells by apoptosis and anoikis. Epigenetic regulation mechanistically includes the methylation and demethylation of DNA, posttranslational histone modifications, interaction with readers, and erasers of these histone modifications. Moreover, epigenetic regulation is mediated by histone variant exchange, non-coding RNAs, which includes micro-RNAs and long-non-coding RNAs (lncRNAs), and changes in short- and long- range higher-order chromatin architecture. The information about changes of epigenetic marks in cancer occurrence and progression can be useful at the level of diagnostics to identify cancer stages and aggressiveness and cancer prognostics. To understand the epigenetic pathways may enable using modulators in order to counteract a non-beneficial epigenetic landscape. Many inhibitors have been developed for proteins with enzymatic activity. Since many epigenetic modifiers possess enzymatic activity, detailed knowledge about the specificity of epigenetic changes in PCa will allow the future use of epigenetic inhibitors for PCa therapy.

The epigenetic regulation of cancer cells by circulating non-coding RNAs is also an important part of oncogenesis and has been recently explored by scientists in the field. It is now well known that non-coding RNAs, including microRNAs and lncRNAs, are present in body fluids secreted in exosomes that are small vesicles about 100 nm in size, and these are thought to be the new way of long-distance communication between cells. lncRNAs are transcripts with regulatory functions shown to be important in the pathogenesis of many types of cancers, including prostate cancer. Interestingly, in PCa cancerogenesis, lncRNAs can act as either oncogenic or tumor suppressor lncRNAs. Some lncRNAs were shown to act within androgen receptor signaling (3, 4). Furthermore, SNPs identified within the lncRNA genes are associated with PCa, suggesting that these have a benefit in tumor evolution. In this Research Topic, the metanalysis of data derived from 474 patients has revealed that the lncRNA SNHG3, SNHG7, NEAT1, PCAT6, and NORAD were overexpressed in PCa associated with lower overall survival and prognostic value. Furthermore, the analysis of clinical samples has indicated that SNHG3 and NEAT1 were overexpressed in PCa bone metastasis compared to primary tumors (Song et al.), suggesting that these lncRNAs have oncogenic activity and may serve as promising predictors for poor prognosis and PCa metastasis. The authors predicted a lncRNA–miRNA network.

Predictions of pathways are becoming more and more important, providing a basis for analysis that must eventually be validated. The prediction of programmed cell death might be important to analyze compounds that activate this pathway in cancer. In their contribution to this Research Topic, Zhao et al. provide a gene set of 12 genes that in combination induces anoikis. Importantly, this anoikis-related gene set signature is prognostic for the biochemical recurrence of PCa patients. Notably, within this gene set, the polo-like kinase 1, PLK1, may act as a regulatory factor as a core gene. Future experiments using the PLK1 inhibitor will provide evidence for its functional role as a therapeutic.

A specific gene signature is suggested to predict the PCa metastatic status of lymph nodes. The contribution by Xie et al. addresses this topic by analyzing RNA-seq splicing events profiles. The analyses include the use of machine learning software classifiers in order to train with stratified five-fold cross-validation. Thereby, out of 333 differentially expressed alternative splicing events that were identified in a prostate adenocarcinoma cohort, a 96 alternative splicing signature was shown to be able to distinguish between lymph node and non-lymph node metastasis. This indicates not only a cancer-specific alternative splicing profile to distinguish between cancer and non-cancer and the immune microenvironment (5) but also an alternative splicing signature that enables differentiation within the tumor evolution towards metastasis. One protein family, as epigenetic acting enzymes, are the Jumanji domain-containing factors that function as lysine and arginine demethylases and in the proteolytic removal of histone tails. The expression profiles of 35 Jumanji domain-containing genes and their association with clinicopathological features were analyzed. Notably, 12 genes were significantly upregulated in PCa. Of these, the ribosomal oxygenase 2 (RIOX2, JMJD10) gene is also significantly associated with disease-specific survival outcomes of PCa patients, with a progression-free interval in patients without lymph node metastasis or with higher Gleason scores. Network analysis has suggested that RIOX2 expression is positively associated indirectly with AR signaling, presumably through c-Myc linking to the epigenetic network. Immune response and inflammation can inhibit tumor progression. Therefore, it seems beneficial that antitumor immunity could have an important role in adjuvant cancer treatment. Single-cell RNA-sequencing analyses in combination with methylation data from 341 PCa and 35 normal prostate tissue specimens identified interleukin-1 receptor-associated kinase 1 (IRAK1) as overexpressed in PCa. IRAK1 has an antiapoptotic effect and is responsible for resistance to radiation-induced tumor cell death. Interestingly, IRAK1 expression is tightly regulated at the epigenetic level. The IRAK promoter is significantly hypomethylated in PCa, suggesting that during PCa cell evolution, IRAK1 is overexpressed, adding resistance to programmed cell death. Therefore, selective IRAK1 inhibitors may be considered a therapeutic option.

A major epigenetic change involving higher-order chromatin structure was described using the co-culture of PCa with monocytes. Cell–cell contact in the tumor microenvironment that also includes non-cancer cells, such as immune cells and cancer-associated fibroblasts, is considered an important feature for cancer progression. The tissues surrounding the cancer cells are composed of fibroblasts, endothelial cells, monocytes, other cells, and extra-cellular matrix. However, in this Research Topic, Alshaker et al. have identified that, without direct cell–cell contact, through the secretion of factors such as exosomes, massive changes in the three-dimensional higher-order chromosome conformations can be induced. The transfer of this information was identified by co-culturing monocytes with PCa cells in transwell chambers separated by a membrane. The authors analyzed chromosome conformations across the whole genome. Interestingly, the co-culture PCa cells showed a change of their chromosome conformation. Moreover, a change of monocyte chromatin conformation was observed, suggesting that the cross-talk between PCa and monocytes can influence each other at the level of the epigenetic higher-order chromatin structure.

Thus, this Research Topic provides novel insights into epigenetic regulation in PCa tumorigenesis.

## Author contributions

AB: Conceptualization, Writing – original draft, Writing – review & editing. MO: Writing – review & editing.
